# Prediction of Bone Mineral Density (BMD) Adaptation in Pelvis–Femur Model with Hip Arthroplasties

**DOI:** 10.3390/jfb12030049

**Published:** 2021-09-03

**Authors:** Abdul Halim Abdullah, Mitsugu Todo

**Affiliations:** 1 School of Mechanical Engineering, College of Engineering, Universiti Teknologi MARA, Shah Alam 40450, Selangor, Malaysia; 2 Research Institute for Applied Mechanics, Kyushu University, 6-1 Kasuga-koen, Kasuga 816-8580, Japan; todo@riam.kyushu-u.ac.jp

**Keywords:** hip arthroplasty, pelvis–femur model, bone remodeling, CT-based images, finite element analysis

## Abstract

The prediction of bone remodeling behaviour is a challenging factor in encouraging the long-term stability of hip arthroplasties. The presence of femoral components modifies the biomechanical environment of the bone and alters the bone growth process. Issues of bone loss and gait instability on both limbs are associated with the remodeling process. In this study, finite element analysis with an adaptive bone remodeling algorithm was used to predict the changes in bone mineral density following total hip and resurfacing hip arthroplasty. A three-dimensional model of the pelvis–femur was constructed from computed tomography (CT-based) images of a 79-year-old female patient with hip osteoarthritis. The prosthesis stem of the total hip arthroplasty was modelled with a titanium alloy material, while the femoral head had alumina properties. Meanwhile, resurfacing of the hip implant was completed with a cobalt-chromium material. Contact between the components and bone was designed to be perfectly bonded at the interface. Results indicate that the bone mineral density was modified over five years on all models, including hip osteoarthritis. The changes of BMD were predicted as being high between year zero and year one, especially in the proximal region. Changes were observed to be minimal in the following years. The bone remodeling process was also predicted for the non-operated femur. However, the adaptation was lower compared to the operated limbs. The reduction in bone mineral density suggested the bone loss phenomenon after a few years.

## 1. Introduction

Bone remodeling effects are a significant issue in promoting the long-term stability of hip arthroplasties. The phenomena of stress shielding and bone loss are a part of the consequences that contribute to the implant’s subsequent loosening and the possibility of bone fractures [1]. Prediction of the bone remodeling mechanism helps to understand and encourage long-term performance. Nevertheless, the process is complicated and challenging as bone behaviour is different in each person. Due to arthritis and arthroplasty, bones modify their architecture and mass to adapt to the new mechanical environment [2].

The presence of the femoral component in hip arthroplasty creates a mismatch material inside the bones. The stiffer materials of the element dominate the mass load and contribute to the stress shielding problems. Hence, the decrease in mechanical load triggers the remodeling process to modify the bone mass and geometry [2,3]. The development of the quantitative bone remodeling simulation method has encouraged advanced studies in predicting the influence of the femoral component [4] and implant material properties [5] on the bone remodeling process.

The risk of bone loss associated with the remodeling process have been continuously investigated in hip osteoarthritis and arthroplasties cases. Several consequences are to be expected from the disease and treatment, including other joint disorders [6] and gait impairment [7]. Kiss [8] reported that the performance of both non-affected and affected limbs was modified due to hip osteoarthritis. The non-affected limb demonstrated a compensation mechanism, while the affected limb had its motion and functional abilities limited [9]. Similarly, the non-operated limb of hip arthroplasty was reported to experience similar consequences [10,11]. The limb was expected to demonstrate greater force and momentum to compensate for the operated (hip arthroplasty) limb.

To the authors’ knowledge, neither computational nor experimental study has been conducted to evaluate the bone adaptation and remodeling effects in the non-operated limb. Therefore, this study aimed to analyse the bone remodeling in both operated and non-operated femur models after hip arthroplasties. Finite element analysis with an adaptive bone remodeling algorithm was applied to forecast the changes in bone mineral density following total hip and resurfacing hip arthroplasty.

## 2. Materials and Methods

**Figure 1:**
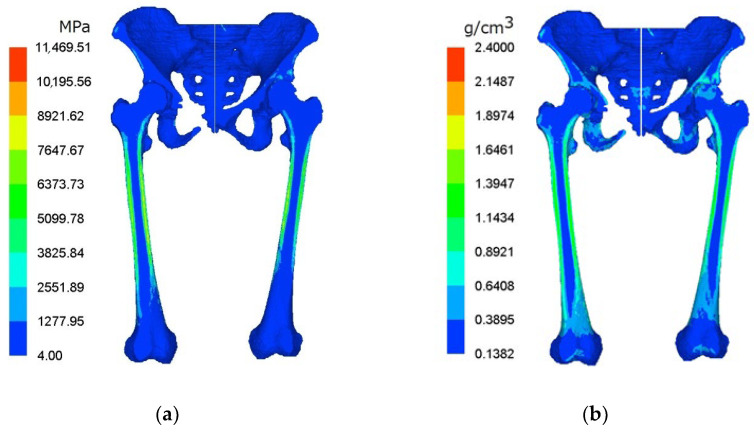
Cross-sectional view of (**a**) young modulus and (**b**) bone mineral density in hip OA pelvis–femur model.

Computed tomography (CT) data images were extracted to develop a three-dimensional pelvis–femur model. The material property of the bone was determined based on the linear relationship between apparent density and the Hounsfield greyscale unit of the CT data [12,13]. Commercial biomedical software, Mechanical Finder (MF) v6.1 (Research Center of Computational Mechanics Inc., Tokyo, Japan), was used to generate the heterogeneous and isotropic material of the lower limbs model. Variations in bone mineral density and young modulus of the pelvis–femur model are illustrated in Figure 1, respectively.

The hip arthroplasties model was constructed by replacing the left hip joint with femoral components. The acetabulum was reamed and replaced with an artificial acetabular cup. For total hip arthroplasty (THA), the femoral head was cut off and inserted with a prosthesis stem. The stem was aligned to fit the femoral canal before inserting the femoral head correctly. The Alumina-on-Alumina type of prosthesis was considered in the analysis to comply with the CAD file provided by the hospital.

**Figure 2:**
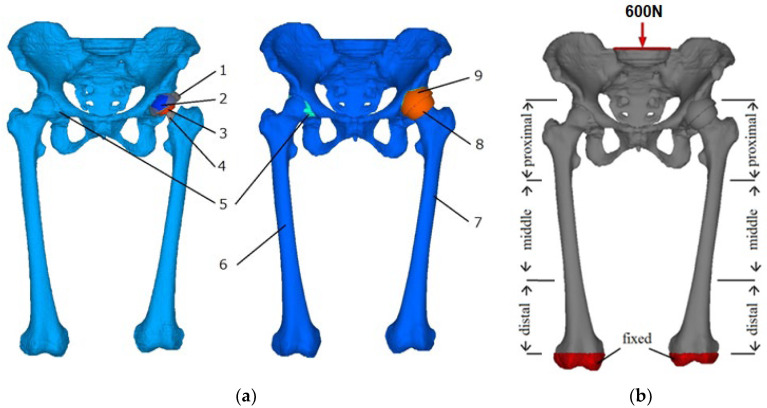
(**a**) 3D model of the pelvis–femur model with THA (left) and RHA (right) on the left limb with (1) acetabular cup, (2) bearing liner, (3) femoral head, (4) prosthesis stem, (5) hip cartilage, (6) right limb, (7) left limb, (8) prosthesis pin, and (9) acetabular cup. (**b**) Boundary condition of quiet standing position and division of different regions in femur model.

Meanwhile, in resurfacing hip arthroplasty (RHA), the femoral head was resurfaced to install the femoral component. Less bone removal was involved in the RHA as compared to the THA model. Descriptions of the lower limbs with the hip arthroplasties model are illustrated in Figure 2a, while the mechanical properties of the femoral components are summarized in Table 1. Interface connection between femoral components and bone was assumed to be perfectly bonded.

An axial distributed load of 600 N was applied in the cross-section of lumbar vertebrae and fixed at the distal end of femoral shafts to estimate the loading distribution, as shown in Figure 2b. The posture in the foot side-by-side position was known to contribute to the structural and functional equivalent of the lower limbs [14,15]. The model was sectioned into three regions, namely proximal (I and IV), middle (II and V), and distal (III and VI) to differentiate affected areas in the remodeling process.

### Bone Remodeling Mechanism

The bone remodeling mechanism was predicted using the bone density life with age-dependent equations developed by Gesso [16,17]. The bone remodeling theory was summarized as

(1) E (t+∆t)=E(t)+α(U(t)−Uo)+β

where α=C ∆t; β=G ∆t; ∆t is time (age) increment; *E* is the young modulus; *U* is the strain energy density; C, *U_o_*, and G are constant, corresponding to sex and t_s_ (stopping age of bone growth). C characterizes the speed of ageing, *U_o_* presents the cortex formation between cortical and cancellous bone while G is the growth constant in the remodeling code. The value of *E* is calculated within the range of

(2) $$E_{\min}\;\leq E\;\leq \;E^{\left(0\right)}\max+\overset{1}{\underset{0}{\int }}G\;dt$$

and

(3) $$E_{\max}^{\left(0\right)}=E_{\max}-\;\overset{ts}{\underset{0}{\int }}G\;dt$$

The relation between bone density, *ρ* (g/cm^3^) and young modulus, *E* (Pa), was specified as

(4) $$\rho =6.414\times 10^{-4}\;\sqrt[{3}]{E}$$

The remodeling sub-program was implemented in the Mechanical Finder software to calculate the relation between bone density and young modulus and predict the bone remodeling mechanism in the pelvis–femur model.

## 3. Results

### 3.1. Remodeling Behaviour in Pelvis–Femur Models

**Figure 3:**
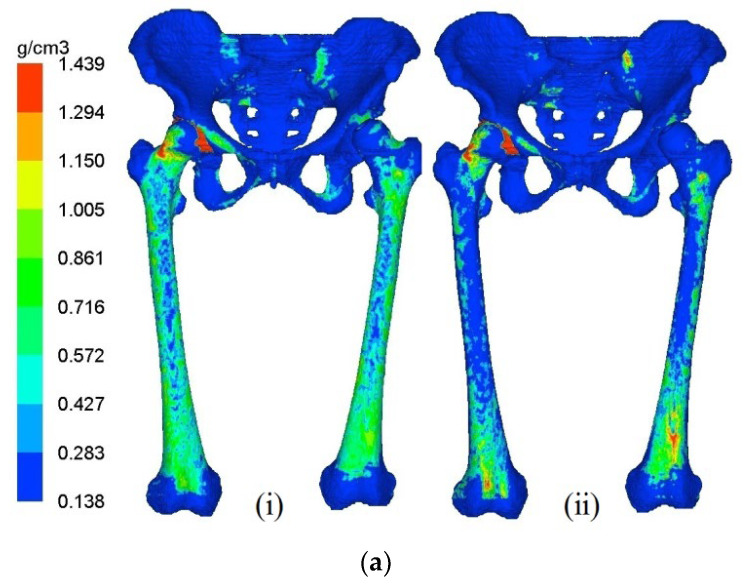

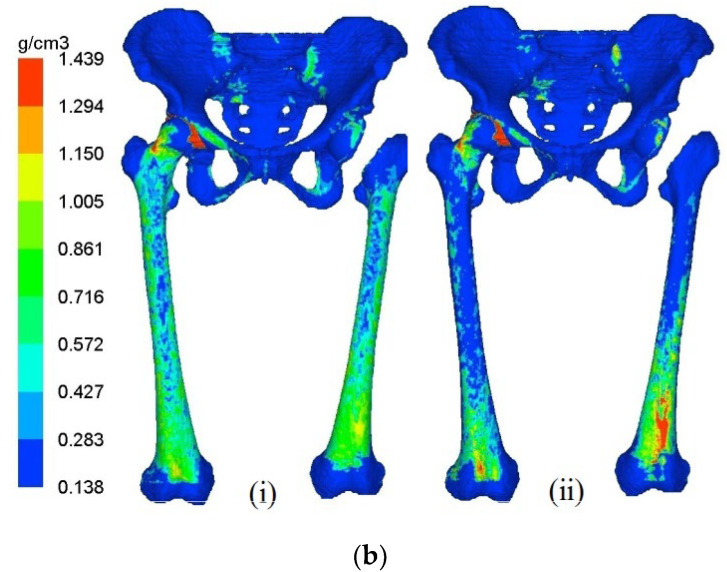
Bone remodeling mechanism of the pelvis–femur model after (**i**) Year 1 and (**ii**) Year 5 for (**a**) RHA and (**b**) THA.

The distribution of bone mineral density in pelvis–femur models at year 1 and year 5 are illustrated in Figure 3a,b for THA and RHA cases, respectively. At year 0 or the initial stage, the bone density distribution was similar in all models except for the bone removal region. The stage of year 1 indicates the changes in bone mineral density (BMD) during the early phase, while year 5 suggests the final condition after 5 years of the remodeling process. The femoral component at the left joint was hidden to observe the remodeling behaviour in the bones only.

The remodeling process was predicted for both right and left femurs for all cases with the proximal region experiencing the most significant changes. Withal, the adaptation was observed to be dominant at the left femur, presenting the hip arthroplasties. The presence of the stiffer material of the femoral components at the respective joint that led to stress shielding effects was believed to lead to the alteration process. The decrease in BMD was also considered to be a phenomenon of bone loss.

### 3.2. Bone Mineral Density (BMD) Adaptation in the Non-Operated Femur

The pattern of BMD adaptation in the right or non-operated femurs over the 5 years was predicted in all models (hip OA, THA, and RHA). The mean value of BMD within the proximal, middle, and distal regions was calculated to estimate the remodeling behaviour.

**Figure 4:**
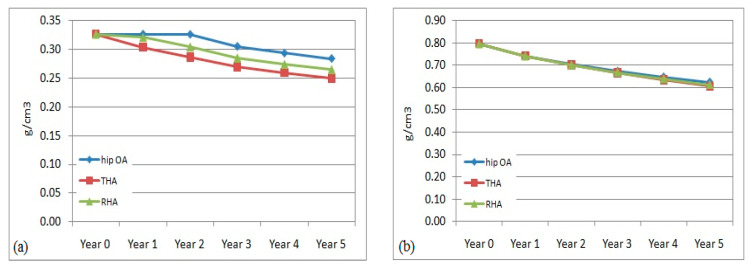

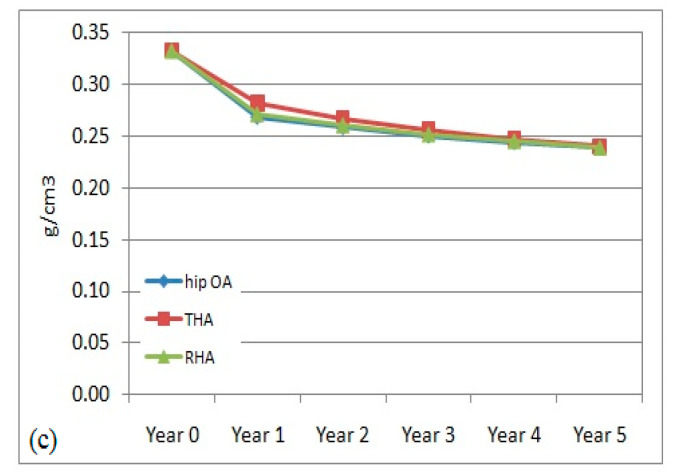
Mean bone mineral density in non-operated femurs in (**a**) proximal, (**b**) middle, and (**c**) distal regions.

The right femur was also affected with bone remodeling and bone loss due to hip arthroplasty at the left femur. The evaluation of the bone density adaptation is illustrated in Figure 4. The reduction in BMD was also observed to occur over the 5 years. In the middle and distal regions, the percentage of BMD reduction was almost similar within all models. The changes were computed to be within 3.5% to 7% at the central region and from, approximately, 1.5% to 5% in the distal region. The reduction pattern was slightly different at the proximal region between the non-affected (hip OA) and non-operated (THA and RHA) models. In the hip OA model, the change started in year 2, while in the non-operated femur the change started immediately in year 1. The difference in the THA model was still higher at 3.5–7% compared to the RHA femur at 1–6% over the 5 years. The findings suggested that a stiffer implant in the operated femur had also affected the remodeling process in the non-operated femur instead of just the operated femur, especially in the proximal region.

### 3.3. Prediction of Bone Loss in Operated Femur

**Figure 5:**
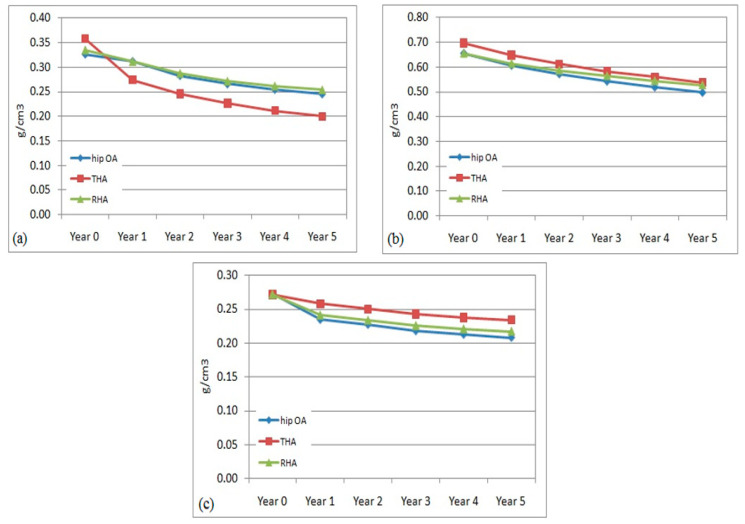
Mean bone mineral density in operated femurs in (**a**) proximal, (**b**) middle, and (**c**) distal regions.

Prediction of bone remodeling in the left femur is illustrated in Figure 5 at different regions. The adaptation process is expected at all regions with a decreased BMD every year. The pattern of bone remodeling in the hip OA and RHA models demonstrated an almost similar pattern and magnitude. The minimum amount of bone removal in the RHA was believed to contribute to the finding. Meanwhile, the value of mean bone density in the THA model was predicted to be lower than the other models in the proximal region. However, it remained higher in the middle and distal regions. The removal of the femoral head for the prosthesis stem and femoral head modified the bone character in the respective region.

**Figure 6:**
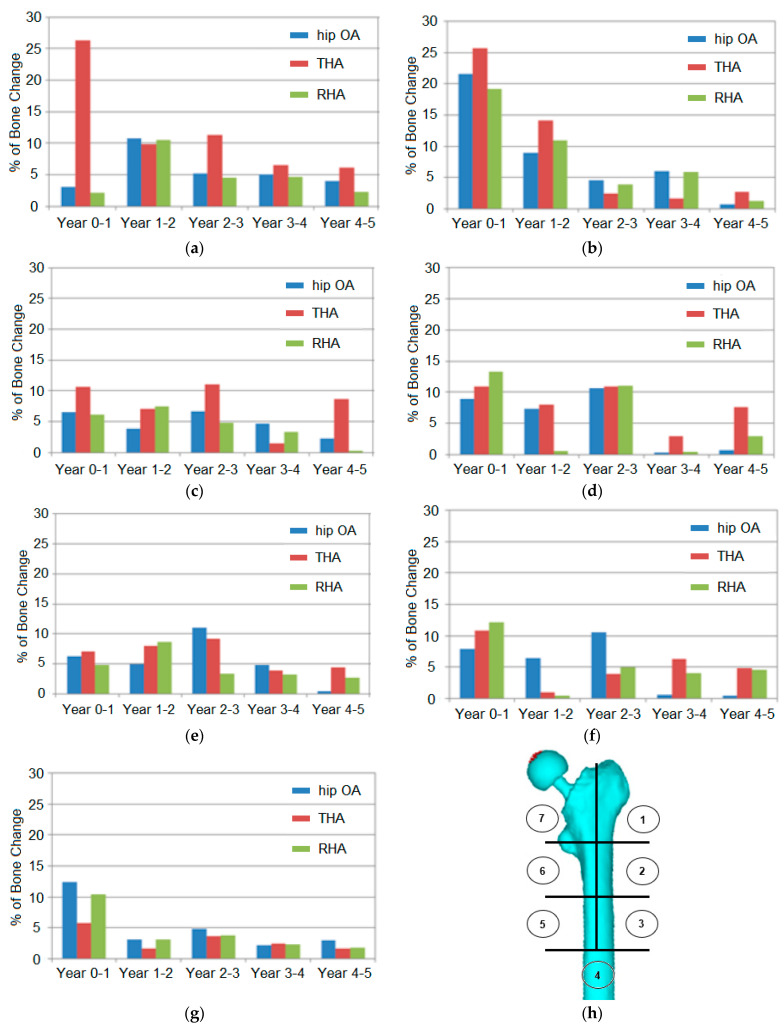
Prediction of bone loss in the operated femur, corresponding to five years, at different Gruen zones. (**a**) zone 7, (**b**) zone 1, (**c**) zone 6, (**d**) zone 2, (**e**) zone 5, (**f**) zone 3, (**g**) zone 4 and (**h**) location of Gruen zones: ①②③④⑤⑥⑦.

The prediction of the bone loss in the hip OA and arthroplasty femurs, corresponding to five years, is explained in Figure 6. The femur was divided into seven areas based on Gruen zones. The designation of Gruen zones is synonymous with THA cases, but the segmentation of regions helps explain bone behaviour in the hip OA and RHA models. The percentage of change was calculated based on the difference of mean BMD between years in the respective region.

The findings indicated that bone loss was expected to happen in all regions and models. The proximal region (zones 1 and 7) was the most affected by bone loss, especially in the THA model. The initial period between years 0 and 1 at zone 1 presented the highest percentage rate (19–26%) in all models. The percentage difference was elevated up to 27% at zone 7 for the THA model. However, the difference was minimal in the other zones at 3–4% only. In the continuing years, the percentage change decreased.

The percentage of BMD change in the THA model was also higher (3–11%) than in the other models (1–6%) in zone 6. The effects of stress shielding in this region were believed to contribute to the findings. Meanwhile, the other regions demonstrated a variety of minimal changes in all models. In zone 2, 3, and 5, the change percentage ranged from around 1% to 10% in all models throughout the years. The lowest changes were calculated in zone 4 with only 2–5%, except in years 0–1. A higher percentage of BMD change in the initial year suggested a critical adaptation and remodeling process.

## 4. Discussion

The finite element simulation contributed to quantifying the changes that occurred in the proximal femur [18]. The computational prediction of bone mineral density (BMD) changes in hip arthroplasty have been continuously investigated [2,18,19], especially in THA cases. Different methods and remodeling algorithms have been developed and improved to support the precision of the estimation [4,16,20].

The prediction of high bone loss in the early stage after THA was consistent with clinical observation [21,22,23,24,25]. Venesmaa et al. [21] reported that the reduction in periprosthetic BMD was within the range from 5% to 18% in all Gruen zones after three months, and the loss continued up to 6 months. Only minor changes in BMD were found from 1 to 5 years after. The most significant bone loss at 25% in the calcar region during the first postoperative year was also almost similar to that predicted in zone 7 of this study. Issues of stress shielding effects, the immediate bone response to the implantation, and bone disuse atrophy were agreed to correlate with higher bone loss at the early stage [19,26,27]. Smaller percentages of BMD reduction in the later period after arthroplasty were projected to be approximately 1% a year [28]. This was expected due to the normal ageing of bone.

Projection of the bone remodeling behaviour in the RHA model was associated with the possibility of femoral neck fractures. As the resurfacing techniques involved the modification of the femoral head and neck, the proximal region of the femur was expected to experience more changes. Willis-Owen et al. [29] reported that the BMD of the femoral neck only decreased in the first three months, and then kept increasing for at least five years. A comparison study between THA and RHA by Kishida et al. [30] also found an increase in BMD in Gruen 1 and 7 following hip arthroplasty, while there was a BMD reduction following total replacement for two years. Similar findings by Smolders et al. [31] indicated an initial BMD reduction for the first 12 months after hip resurfacing, followed by an increment up to 105.2%. The findings concurred with this study, which predicted higher bone loss at the initial stage.

However, the increment in BMD in the continuing years, as reported in other studies, [29,30,31] was in contrast to this study. A minimal reduction was expected in the following years, similar to that projected in the hip OA and THA femurs. In this work, the bone was extracted from an elderly patient, while the previous subject was a young and active patient. The bone remodeling process reduced the strain concentration in hip resurfacing, increasing the initial risk of femoral fracture neck [32], especially in young patients. Ideally, the RHA procedure should not be suggested for elderly patients due to their poor bone stock [33].

The reduction in BMD in the hip OA model was expected as a reduced hardness of trabecular bone from the femoral head was found in the severe OA [34]. Additionally, the shape deformities and the change in microstructure of the bone could also have contributed to the bodyweight loading adaptation and remodeling process [35]. Similarly to the arthroplasty model, the BMD in the hip OA model decreased in the early phase and minimally in the succeeding years due to ageing. In OA, ageing was considered a primary risk due to the deprivation of standard bone structure and the accretion of bone microdamage [36]. In addition, the reduction in BMD can partly be clarified by patient-related factors such as decreased mobility [37]. The difference in structure and bone density between healthy OA and arthroplasty femurs leads to the imbalance and instability on both right and left femurs. Instead of the stress adaptation after arthroplasty [38], the remodeling process’s diversity over the years was also suggested as a reason for gait impairment and stability. The findings parallel the rehabilitation study, which indicated that gait adaptation occurs in non-operated limbs but not in operated femurs [39].

Several other factors were associated with the bone loss and remodeling processes, such as BMI, age, and preoperative BMD on femoral bone loss [21]. The morphological and bone mass changes occurred in the immediate postoperative period [18] to adapt to the new biomechanical environment [2]. The bone mass increased in the loading areas while decreasing in unloaded areas [20]. The poor bone quality became a risk to socket survival, while the poor bone structure was a risk for the prosthesis survival [40].

Several limitations of the study were identified. The study was conducted based on single CT-data images. Multiple CT data and analysis will provide more convincing findings, especially in evaluating a cohort of cases. Next, the study did not count muscle response such as abductor force, which may contribute to gait balancing and stability [41,42]. The time frame allocated for the sub-program ranged across years, while clinical observations varied across months. A shorter scale period of remodeling prediction will provide a proper comparison.

## 5. Conclusions

Prediction of bone remodeling behaviour in the pelvis–femur model was presented with the changes in bone mineral density. Both operated and non-operated femurs were expected to experience bone remodeling corresponding to five years of prediction. The proximal regions of all models were identified to experience the most dramatic bone loss. Additionally, the initial phase between years 0 and 1 demonstrated the highest BMD changes. The computational findings also suggested that the THA model experienced greater bone loss than the RHA and hip OA models.

## Figures and Tables

**Table 1:** Mechanical properties of total hip and resurfacing hip arthroplasties.

Type	Model	Material	Elastic Modulus, E (GPa)	Poisson Ratio, v
THA	Acetabular cup	Ti-Alloy	114	0.34
	Bearing liner	Alumina	370	0.23
	Femoral head	Alumina	370	0.23
	Prosthesis stem	Ti-Alloy	114	0.34
RHA	Acetabular cup	Co-Chrome	230	0.30
	Prosthesis pin	Co-Chrome	230	0.30

## Data Availability

The data presented in this study are available on request from the corresponding author.
